# Enteropathogenic Infections: Organoids Go Bacterial

**DOI:** 10.1155/2021/8847804

**Published:** 2021-01-07

**Authors:** Viktoria Hentschel, Frank Arnold, Thomas Seufferlein, Ninel Azoitei, Alexander Kleger, Martin Müller

**Affiliations:** Department of Internal Medicine I, Ulm University, 88079 Ulm, Germany

## Abstract

Enteric infections represent a major health care challenge which is particularly prevalent in countries with restricted access to clean water and sanitation and lacking personal hygiene precautions, altogether facilitating fecal-oral transmission of a heterogeneous spectrum of enteropathogenic microorganisms. Among these, bacterial species are responsible for a considerable proportion of illnesses, hospitalizations, and fatal cases, all of which have been continuously contributing to ignite researchers' interest in further exploring their individual pathogenicity. Beyond the universally accepted animal models, intestinal organoids are increasingly valued for their ability to mimic key architectural and physiologic features of the native intestinal mucosa. As a consequence, they are regarded as the most versatile and naturalistic *in vitro* model of the gut, allowing monitoring of adherence, invasion, intracellular trafficking, and propagation as well as repurposing components of the host cell equipment. At the same time, infected intestinal organoids allow close characterization of the host epithelium's immune response to enteropathogens. In this review, (i) we provide a profound update on intestinal organoid-based tissue engineering, (ii) we report the latest pathophysiological findings defining the infected intestinal organoids, and (iii) we discuss the advantages and limitations of this *in vitro* model.

## 1. Introduction

The human intestinal tract can be affected by a myriad of infectious diseases ultimately impairing the intestinal mucosa's capability of regulating the net water absorption, electrolytes, and nutrients, while retaining its function as a physical barrier. In particular, infections of bacterial, viral, or protozoan origin rate among the most common causes of diarrheal diseases, both in resource-rich and -poor countries. Such infections are frequently linked to low hygienic standards and improper handling of food and drinks, as well as occupational exposure to domestic livestock [1]. Often, patients experience watery diarrhea as the only or leading symptom at a varying level of severity, optionally accompanied by hematochezia, abdominal cramps, emesis, or febrile temperatures [2]. Most of the cases of intestinal infectious diseases usually resolve spontaneously or can be treated with exclusively supportive measures such as rehydration and correction of serum electrolytes. However, health care systems across the globe continue to face recurrent infectious disease outbreaks, mostly resulting from the coincidence of several contributory factors: increased transmission rates due to crowded living conditions, limited access to sanitation, and a shortage of public health institutions to put effective prevention and control measures in place [3]. Particularly in developing countries, poor planning and/or poor implementation of health policies and programs negatively impacts on the availability, accessibility, affordability, and sustainability of a healthcare service. In these countries, it is estimated that around 10% of hospitalized patients acquire an infection during their stay. This is intensified by inaccurate diagnoses, medication errors, inappropriate or unnecessary treatment, and inadequate or unsafe clinical facilities or practices. For example, the inappropriate administration of antibiotics over the past decades has led to an accumulation of highly resistant and difficult-to-treat bacterial pathogens [4]. This worrying development has prompted increased efforts both to devise alternative therapeutic strategies and to constantly deepen our current knowledge about pathogen-specific transmission routes, modes of intracellular replication and propagation, and reactive defense mechanisms of the infected host cell. In the past, intestinal cell lines were widely used to construct *in vitro* models of human infectious diseases and to gain insight into their molecular pathomechanisms. However, compared to nontransformed intestinal epithelium, established cell lines usually originate from a cancerous clone with abnormal growth and differentiation behavior as well as altered physiological features, which substantially limit their potential to recreate *in vivo* conditions.

**Figure 1:**
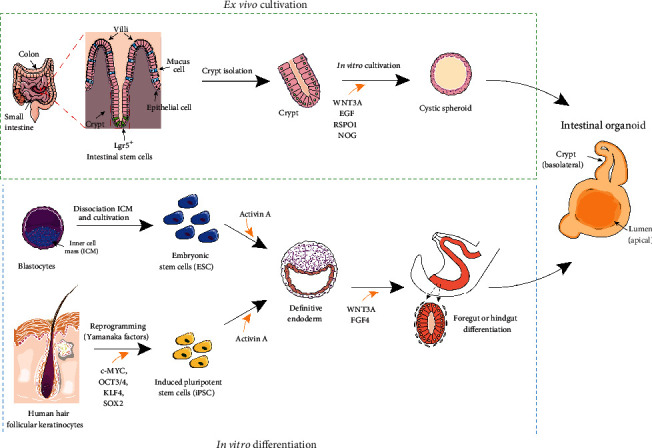
Generation of intestinal organoids from multipotent intestinal stem cells (ISC), embryonic stem cells (ESC), and induced pluripotent stem cells (iPSC). The protocols illustrated above are routinely applied in our laboratories.

In recent years, intestinal organoids have emerged as a promising tool, allowing researchers to establish long-lasting stem cell-based cultures dedicated to the intestinal epithelium in the absence of feeder cells. Cell proliferation and the growth of organoid culture systems are thereby sustained by adding appropriate stem cell niche factors to the culture medium. Intestinal organoids may emanate either from pluripotent stem cells of embryonic origin (ESC) or be reprogrammed by overexpression of pluripotency genes (c-MYC, OCT3/4, KLF4, SOX2) in somatic cells (iPSC). Alternatively, they may be derived from multipotent organ-committed leucine-rich repeat-containing G-protein coupled receptor 5 + (Lgr5+) crypt columnar base intestinal stem cells (ISC) (Figure 1). With regard to the latter, suitable tissue material can be obtained either from human donors undergoing endoscopy-guided biopsy or surgical resection or can be extracted from the whole murine intestine of sacrificed animals. The foundation of this fascinating tissue engineering technique was laid by Hans Clevers and his research group, who for the first time allowed the implementation of a robust 3D culture system of the intestinal epithelium originating from a single ISC [5]. Reproducible cultivation methods, amenability to experimental genetic manipulation, and conserved primary cell biology have all contributed to predestine intestinal organoids as an extremely useful tool to model host-pathogen interactions in human-relevant diseases. Embedding in an extracellular matrix-like scaffold and supplementation with the essential niche factors, epidermal growth factor (EGF), Noggin, R-Spondin 1, and Wnt3a drive proliferation and asymmetric division of the ISC to yield the rapidly cycling transit amplifying compartment. Next, the already lineage-committed progeny starts to form immature spheroids which are subsequently transformed into mature intestinal organoids with distinct crypt-villus compartmentalization [5, 6]. The luminal surface facing the inside is lined by a monolayer of polarized columnar epithelial cells which recapitulate the diversity of highly differentiated intestinal cell types typically encountered throughout the intestinal tract. Absorptive enterocytes account for the most prevalent cell type and are principally engaged in the regulation of water and electrolyte balance as well as the absorption of nutrients [7]. As a prerequisite for charge- and size-selective permeability, paracellular diffusion is restricted by an intercellular network of tight junctions. Besides absorptive enterocytes, the intestinal epithelium is interspersed with the following highly specialized cell types. (i) Goblet cells produce a viscid mucus rich in complex glycoproteins (mucins) which functions as a physical barrier between the host epithelium and the luminal microbiota. Goblet cells are perceived as an adjunct to innate immunity, as they produce various antimicrobial proteins such as angiogenin 4 [8], chemokines, and cytokines [9–12]. (ii) Paneth cells originate from and remain in the close vicinity of the ISCs, whose capacity for self-renewal largely depends on the juxtacrine secretion of the growth-promoting niche factors, namely, transforming growth factor, EGF, and Wnt3a from the Paneth cells. Additionally, they support local immune defense by excreting antimicrobial peptides such as lysozyme and *α*-defensins/cryptdins [13, 14]. (iii) Microfold (M) cells are a specialized cell type of the follicle-associated epithelium (FAE) responsible for luminal antigen sampling and trafficking to the underlying lymphoid tissue, thus contributing to mucosal immune surveillance [15]. Under steady-state conditions, the occurrence of this rare cell type is confined to the FAE, where its differentiation mainly depends on the receptor activator of nuclear factor-*κ*B (NF-*κ*B) ligand exclusively secreted by the underlying subepithelial stromal cells [16, 17]. (iv) Tuft cells represent another rare epithelial cell lineage which has been implicated in assisting innate lymphoid cells (ILC) to fight helminthic infections by supplying interleukin (IL) 25. Conversely, exposure to IL 13 derived from activated ILC has been shown to induce tuft cell hyperplasia [18, 19]. (iv) Another epithelial cell subpopulation is represented by the numerically small entity of enteroendocrine cells, among which the enterochromaffin cells constitute the most abundant cell type [20]. Their principal secretory product, serotonin, functions as a regulator of coordinated propulsive gut motility and intestinal fluid secretion [21, 22].

**Figure 2:**
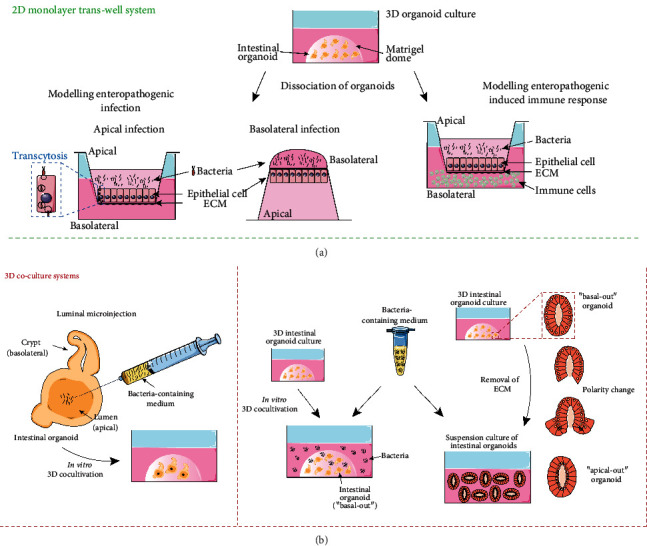
*In vitro* modeling of enteropathogenic infection. (a) 2D intestinal coculture models: bacteria are seeded onto the apical or basolateral surface of the intestinal epithelial monolayer (adapted from: Ranganathan et al., 2019 [38], Koestler et al., 2019 [39]). Optionally, immune cells are added to the basolateral compartment of infected intestinal epithelium (adapted from: Noel et al., 2017 [31], Karve et al., 2017 [49]). (b) 3D intestinal coculture models: bacteria are either introduced into intestinal organoids via luminal microinjection (adapted from: Karve et al., 2017 [49]) or added to the culture medium of “basal-out” or “apical-out” intestinal organoids (adapted from: Co et al., 2019 [59]).

A considerable contribution to early immune response is made by the heterogeneous epithelial cell population of the intestine arguing in favor of the use of intestinal organoids as a stand-alone *in vitro* system for modeling enteric infections (Figure 2). The host immune response is further shaped by various local immune effector cells which can optionally be integrated into the organoids to achieve a more truthful adaptation to *in vivo* conditions. Within recent years, the primary cell-based origin of organoids and their versatility in many fields of application has encouraged the establishment of a series of infection models collectively adding to the pathophysiological understanding of clinically relevant human enteropathogens. The approaches addressed in this review illustrate the latest achievements in generating pathogen-specific intestinal co-cultures for advanced disease modeling and drug screening and outline particular results that have been ascertained (Table 1).

## 2. Bacterial Enteropathogens and Their *In Vitro* Replicas

### 2.1. *Vibrio cholerae*

Cholera is a diarrheal disease affecting mainly malnourished patients in resource-poor countries with reduced access to clean water and inadequate sanitation. The majority of epidemic outbreaks are caused by the serogroups O1 and O139 of the *Vibrio cholerae* (*V. cholerae*) bacterium, whose pathogenicity relies on the production of AB5 cholera toxin (CT). The toxin consists of an A subunit localized at its core, which is surrounded by a pentameric B subunit [23]. The B subunit encompasses an anchoring element with high affinity to the ganglioside molecule GM1. Despite its sparse expression on the host enterocyte surfaces, GM1 is considered a crucial receptor for CT [24]. It has been shown to promote endocytotic absorption of the holo-complex toxin into the host cell where the A subunit triggers adenylate cyclase activity. This results in augmented intracellular levels of the second-messenger molecules cyclic adenosine monophosphate (cAMP) and cyclic guanosine monophosphate (cGMP). Subsequent activation of principal ion selective transport channels, such as the cystic fibrosis transmembrane conductance regulator (CFTR), leads to a dramatic rise in the luminal secretion of chloride followed by a passive efflux of water [25]. The cytotoxic effect can be reliably reproduced *in vitro* by the exposure of intestinal organoids to CT causing a dose-dependent quantifiable enlargement of the organoid volume. For the first time, the intestinal organoid-based swelling assay was validated as a preclinical screening tool for multivalent CT inhibitors by Zomer-van Ommen et al. [26]. By employing human rectal organoids, Haksar et al. not only identified a range of efficacious and at the same time cost-effective compounds featuring metanitrophenyl *α*-galactoside, a well-known ligand to CT [27] but also different polymer organic scaffolds derived from linear polyacrylamide, dextran, and hyperbranched polyglycerol. All compounds tested proved to inhibit CT attachment to and entry into the intestinal cells in an equipotent manner compared to synthetically produced GM1 oligosaccharide [28]. To mimic the fecal-oral infection route of *V. cholerae* and create a physiological model of enteric disease, Kane et al. used intact bacteria for microinjection into the lumen of iPSC-derived small intestinal organoids [29].

### 2.2. Enterotoxigenic *Escherichia coli*

Enterotoxigenic *Escherichia coli* (ETEC) is one of the leading causes of the usually self-limiting traveler's diarrhea and sudden-onset diarrheal illness in areas with low hygienic status. It is mainly caused by the secretion of the heat-labile and heat-stable toxins (LT and ST) which display structural similarity to the CT [30, 31]. Effective colonization of the mucosa, allowing immediate toxin delivery to the intestinal epithelium, is optimized by plasmid-encoded adhesive fimbriae and adhesins. Among the latter, EtpA, a high molecular weight adhesin, has been assigned a key role in promoting colonization of the host epithelium [32, 33]. This adhesin molecule attracted attention when it became evident that human volunteers challenged with oral ingestion of ETEC strain H10407 suffered from diarrhea of substantially varying severity, suggesting the influence of at least one host factor on disease manifestation [34, 35]. Large-scale glycan assays probed with recombinant EtpA revealed preferential binding to N-acetylgalactosamine, a terminal sugar residue pertaining to blood group A. To create a model closely resembling *in vivo* conditions, small intestinal organoids from human donors of each major AB0 blood group were incubated with purified EtpA and EtpA-expressing wild type (WT) H10407 ETEC, respectively. In both cases, epithelial cells bearing blood group A glycoproteins were recognized with higher affinity compared to small intestinal organoids derived from blood group B or 0 donors, suggesting the role of EtpA as a pathovar-specific lectin. In accordance with this finding, adhesion of EtpA-mutant ETEC to blood group A small intestinal organoids occurred more hesitantly compared to EtpA-expressing WT H10407 ETEC. Intracellular levels of cAMP, reflective of toxin-dependent adenylate cyclase activity, were significantly reduced in EtpA-mutant-infected small intestinal organoids, while production of ST did not differ between both groups. These findings indicated that EtpA in the capacity of a pathovar-specific lectin ensures stable binding preferentially to blood group A-glycosylated epithelial surfaces, thereby rendering toxin delivery more efficient [36].

A complex *in vitro* co-culture involving ETEC strain H10407 and peripheral blood monocyte-derived macrophages is aimed at modeling the host innate immune response to an enteric infection (Figure 2). Human small intestinal organoids converted into a confluent monolayer were inoculated with bacteria on their apical surface to imitate the luminal portal of entry. It could be noted that phagocytic activity of macrophages led to an efficient internalization of bacteria, while at the same time, infection-related impairment of the epithelial barrier function was partially restored in the presence of macrophages [37]. This constellation is consistent with previous findings that upon migration into intestinal lamina propria tissue, peripheral blood monocytes differentiate into resident macrophages with an anergic phenotype but preserve transepithelial antigen-scavenging and bactericidal properties [38].

### 2.3. *Shigella flexneri*

*Shigella* sp. rank among the most common causes of infectious diarrhea, especially in debilitated and immunocompromised persons in developing countries. The genome of *Shigella*, a gram-negative nonmotile enterobacterium, is known to harbor a set of virulence factors including *Shigella* enterotoxins 1 and 2 (ShET 1 and 2) and Shiga toxin (Stx), encoded by the chromosomal DNA and the virulence plasmid, respectively. ShET 1 ultimately results in an increased luminal secretion of ions and water by the enterocytes [39], while ShET 2 is involved in regulating secretion of the proinflammatory cytokine IL 8 by the intestinal epithelium [40]. By contrast, *Shigella dysenteriae-*exclusive *Stx* mediates the attachment of the bacterium to the endothelium of the intestinal vasculature. This results in occlusive ischemia, which is further exacerbated by inadequate activation of platelets [41, 42]. Prior to the actual event of invasion, *Shigella* sp. initiates the production of an adhesive biofilm induced by prolonged exposure to bile salts and glucose during small intestinal passage. Adherence analysis in human colon-derived organoids infected with *Shigella flexneri* (*S. flexneri*) revealed the emergence of adhesive structures contacting the host epithelial cell [43]. Basic characteristics of host cell infection with *S. flexneri* were captured in an organoid-derived monolayer model originating from different sections of the human intestine [44]. To gain access to the basolateral epithelial compartment, *S. flexneri* enforces its own transcytosis via M cells of the ileum- and colon-associated FAE. Human ileum organoids pretreated with the NF-*κ*B-inducing ligand tumor necrosis factor (TNF) *α* to specifically expand the M cell population prior to infection with *S. flexneri* serotype 2a strain 2457T, yielded significantly higher numbers of intracellular bacteria than conventionally grown organoids [44]. Inoculation of either the apical or basolateral epithelial surface with pathogenic *S. flexneri* strain 2457T or mitigated plasmid-cured noninvasive derivative strain 4243A confirmed preferential access across the basolateral epithelial membrane. This invasion route proved to be far more frequented by the virulent *S. flexneri* strain 2457T [44]. The same mechanism of entry was observed in a similar experimental design with human colon-derived organoids using the identical *S. flexneri* strain [45]. Additionally, intracellular mobility of *S. flexneri* hijacks the cytoskeleton of the host cell to form long actin polymers [39]. This process has been reported to be essential for the further cytosolic dissemination of *S. flexneri* to neighboring epithelial cells [46]. Assessment of the epithelial immune response in *S. flexneri*-infected intestinal organoids revealed a transcriptional upregulation of IL 8, TNF *α*, interferon (IFN) *β*, and TNF *α*-induced protein 3 (TNFAIP3), which are largely associated with the NF-*κ*B-mediated inflammation signaling pathways [44, 45]. Furthermore, the infected epithelium expressed higher levels of the intestine-specific mucin (MUC) 2 [44, 45]. According to the authors, this finding has so far remained equivocal as it may either signify a protective host response to reinforce the mucus' barrier function or mirror a subversive effect to modify mucus composition to accommodate the pathogen's requirements [44].

Another field of application of intestinal organoids has evolved with the experimental usage of bacteriophages to specifically fight *Shigella* infections. Frequent administration of antibiotics has given rise to the emergence of resistance plasmids, calling for an alternative therapeutic approach. Bacteriophages refer to viruses exclusively infecting and replicating in bacterial cells. A prominent feature refers to their property to target distinct bacterial species or even specific strains within a species, whereby the phages pursue either a lytic (exploitation of the host translation machinery with subsequent cell death and release of new phages) or a lysogenic replication strategy (mere incorporation of the phage DNA into the host genome, host cell remains unscathed). A therapeutic trial with bacteriophages to fight *Shigella* infection was conducted by Llanos-Chea et al. in both the human colorectal cancer cell line HT-29 and intestinal organoids [47]. Human intestinal organoids were inoculated with several *Shigella* sp. including *S. flexneri* serotype 2a strain 2457T. Subsequent co-incubation with the bacteriophage *φ*2457T demonstrated an efficient clearing of infection with *S. flexneri* serotype 2a strain 2457T, reflected by diminished bacterial recovery rates for both adherence and invasions assays [47].

### 2.4. Enterohemorrhagic *Escherichia coli*

Enterohemorrhagic *Escherichia coli* (EHEC), a gram-negative, rod-shaped enterobacterium, is a human pathogenic strain associated with food-borne colitis with occasional outbreaks of bloody diarrhea [48]. EHEC serotype O157:H7 is commonly responsible for a particularly aggressive disease course involving the hemolytic-uremic syndrome. It is precipitated by the Shiga-like toxins (Sltx) 1 and 2 and characterized by a non-immune hemolytic thrombotic microangiopathy of the kidneys, ultimately leading to acute renal impairment [49]. To probe the initial steps of epithelial invasion, human colon-derived organoids converted into an epithelial monolayer were apically infected with the Sltx-negative EHEC O157:H7 strain EDL933 and mutants deficient for the virulence factors StcE or EspP [50]. StcE refers to a zinc metalloprotease engaged in cleaving the protective layer of mucin glycoproteins to facilitate the attachment of bacteria to the intestinal epithelium [51]. However, infection with a StcE-deficient EHEC strain did not result in impaired destruction of the mucus layer previously reported for EHEC, suggesting an alternative mucus-depleting pathomechanism. EspP is a member of the family of high molecular weight serine protease autotransporters shared among several *Enterobacteriaceae* species and plays a critical role in the disruption of actin-bound cytoskeletal proteins in the host cell [52]. The authors of this study demonstrated that EspP promotes proteolytic reduction of the brush border resident protein protocadherin 24, leading to subsequent effacement of the microvillar bridges which is considered to be a hallmark of EHEC infection [53]. Interestingly, human colon-derived organoid-based *in vitro* studies revealed that EspP can also functionally act as an enterotoxin by triggering aberrant ion currents independent of CFTR activity, potentially contributing to diarrheal symptoms [54].

Human intestinal organoids *in vitro* differentiated from the H1 human embryonic stem cell line were used by Karve et al. to emulate an enteric infection with the Sltx-producing EHEC strain O157:H7 (STEC) [55]. In accordance with the preceding study, gradual disruption of the epithelial lining in conjunction with a perturbed actin cytoskeleton occurred after luminal microinjection of STEC accompanied by the intimate apposition of pathogens to and eventually breaching of the intestinal epithelial barrier. Consistent with the natural course of the infection, culture conditions allowed detection of the host cell-derived burst of reactive oxygen species and reactive induction of Sltx by STEC. The host immune response was characterized by upregulated epithelial expression of the chemokines IL 1*β* and IL 18 and recruitment of co-cultured polymorphonuclear cells from the periphery into the organoids [55].

### 2.5. *Salmonella enterica*

*Salmonella enterica*, a gram-negative facultative anaerobe, rod-shaped, motile bacillus, which is ranked among the most common causative agents of food-borne diarrheal illnesses, is equipped with an ample armory of virulence factors to facilitate attachment, invasion, replication, and evasion of the host immune detection [56]. Preliminary data unveiled a predilection of *π*-class Std fimbriae encoded by the Std operon of *Salmonella enterica* serotype *typhimurium* (*S. typhimurium*) for binding terminal *α* 1,2-fucose residues [57]. This enzyme catalyzes the addition of fucose sugar to host membrane-bound glycans crucial to the expression of ABH and Lewis histo-blood group antigens on mucosal membranes and in body fluids [58]. This adherence strategy has been further corroborated by *in vitro* studies on intestinal organoids grown from *α* 1,2-fucosyl transferase 2 WT mice. Ileum- and colon-derived organoids were inoculated with a Std fimbriae-expressing apathogenic *Escherichia coli* strain which preferably bound to fucosylated cells [59]. Furthermore, Rouch et al. demonstrated that in human small intestinal organoids, *S. typhimurium* selects M cells as their preferred portal of entry [60]. Furthermore, if applied in highly infective doses, it induces an additional transdifferentiation of enterocytes into M cells [60]. To gain access to and travel inside the host cell, *Salmonella* sp. have been shown to exercise control over the intracellular signaling pathways involved in cytoskeletal rearrangement processes. One such mechanism deployed by *Salmonella* sp. aims at manipulating the host GTP-ases Cdc42, Rac1, and RhoG via secreting effector proteins into the host cell to activate the Arp2/3-complex. This central element steering the actin filament assembly is required for the formation of lamellipodia and membrane ruffles, providing intracellular mobility for and permitting the ingress of pathogens into the cell [61–63]. Invasion of the host cell by *Salmonella* sp. appears to be made through the apical transmission route. This finding was confirmed in human small intestinal organoids whose cell polarity had been reversed by depriving them of a matrix scaffold after maturation. *S. typhimurium* added to organoids with reversed polarity (“apical-out”) and conventionally grown organoids (“basal-out”) as well as organoids with a mixed phenotype preferentially penetrated the host cell from the apical surface. Upon intracellular replication of *S. typhimurium*, the infected host cell is usually shed into the luminal space, as has been previously reported in the human colon cancer cell line Caco-2 and murine primary intestinal cells [64]. This exit strategy was reproduced in “apical-out” small intestinal organoids that had been infected with *S. typhimurium*. Hereby, bacteria were detected both within actively extruding epithelial cells and fully extruded epithelial cells [65]. Further investigations centered on the prominent role of the host cell cytoskeleton for the intrusion and intracellular mobility of *Salmonella* sp. were conducted on human ileum-derived organoids inoculated with *Salmonella enterica* serotype *typhi* strain Ty2 (*S. typhi*). Transmission electron microscopy images confirmed the presence of cytoskeletal protrusions suggestive of microvilli dissolution and cytoplasmic reorganization as observed in whole tissue biopsy samples. It could be demonstrated that upon pre-incubation of the organoids with an actin or microtubule inhibitor, the cytoskeleton-dependent mechanism of invasion of *S. typhi* was efficiently disabled [66]. In line with that, intestinal organoids derived from murine ileum and jejunum displayed significant decomposition and downregulation of the tight junction-defining protein Zonula occludens protein 1 following colonization with *S. typhimurium* strain 14028S [67]. Furthermore, in this study, particular interest was vested in examining the epithelial immune response which was characterized by increased NF-*κ*B signaling and consecutive upregulation of the downstream proinflammatory cytokines IL 2, IL 4, IL 6, TNF *α*, and IFN *γ* [67]. Similar results were obtained from an iPSC-based intestinal organoid model infected with *S. typhimurium* strain SL1344 [68]. Gene expression analysis of the host epithelium displayed a preponderance of proinflammatory cytokines such as IL 8, IL 1*β*, IL 23A, TNF *α*, and CXCL 2 but also of the goblet cell-associated genes encoding glucosaminyl-N-acetyl-transferase 3 and MUC 2, suggesting a reactive proliferation of the goblet cell population [68]. By contrast, commensal bacteria colonizing the gut lumen have been assigned an overall protective role by reducing mucosal inflammation and restoring intestinal homeostasis in invasive enteric infections. The integrity of small intestinal organoids challenged with *S. typhimurium* strain SL1344 rapidly deteriorated unless pretreated with the probiotic *Lactobacillus acidophilus* ATCC4356 (*L. acidophilus*). Furthermore, addition of *L. acidophilus* to the organoids caused a reversal of Wnt3 and Toll-like receptor 2 and 4 upregulation, which had been precipitated by *S. typhimurium* infection [69]. Based on the authors' opinion, these results implied an *L. acidophilus*-induced correction of crypt hyperproliferation towards physiological levels and reduced susceptibility towards inflammatory stimuli [69].

### 2.6. *Listeria monocytogenes*

*Listeria monocytogenes* (*L. monocytogenes*) is a gram-positive, motile, rod-shaped bacterium causing food-borne diarrheal illness in immunocompetent persons but triggering septicemia and meningitis in immunocompromised patients and neonates [70]. Previous reports indicated that *L. monocytogenes* preferably traverses the intestinal epithelium both through goblet cells and M cells at the Peyer's patch level [71, 72]. Recently introduced by Roodsant et al. as an equivalent novel organoid culture model, human fetal tissue-derived intestinal organoids were plated as a monolayer and apically inoculated with *L. monocytogenes* which predominantly colocalized with MUC 2-positive goblet cells [73]. Furthermore, it was noted that the fluorescent staining signal for actin became weaker in the apical region of infected cells [73]. This finding might be linked to the property of *L. monocytogenes* to rearrange the host cell's actin into so-called “comet tails” to facilitate intracellular mobility, as previously reported by Co et al. in human small intestinal organoids [65]. *L. monocytogenes*' predilection sites of entry in enterocytes are not limited to specific cell types but also include areas with ubiquitously expressed adhesion protein E-cadherin and the hepatocyte growth factor receptor-associated tyrosine kinase Met. Both are exploited as target receptors by the two major invasion proteins In1A and In1B, respectively, to initiate the endocytotic uptake of *L. monocytogenes* into the host epithelium [74–76]. Under identical experimental conditions as previously described by Co et al., “apical-out,” “basal-out,” and mixed-polarity human small intestinal organoids were inoculated with *L. monocytogenes*. It was demonstrated that *L. monocytogenes* more frequently adhered to “basal-out” small intestinal organoids and spots of exposed basolateral space in “apical-out” intestinal organoids [65]. Such an uneven distribution pattern is attributed to the basolateral localization of E-cadherin and Met and particularly gains in importance at the villus tip, where the epithelial lining is occasionally interrupted by the expulsion of apoptotic enterocytes into the lumen. In the early phase of enteric infection with *L. monocytogenes*, the epithelial segment adjacent to the Peyer patches has been suggested to occupy a central position in initiating an efficacious host immune response [77]. Additionally, it has been implicated in modulating intestinal epithelial homeostasis by inducing acceleration of intestinal villus epithelium renewal and a decline in goblet cell numbers to lock down one potential portal of entry for *L. monocytogenes*. In an intestinal organoid-based model, it was demonstrated that for the induction of epithelial cell proliferation, phosphorylation of both signal transducer and activator of transcription (STAT) proteins STAT1 and STAT3 is mandatory [78]. Intriguingly, STAT1 and STAT3 appear to exert opposing cellular functions with regard to cell cycle regulation, survival signaling, and tumor immunity [79]. *In vitro* activation of the respective STAT proteins could be elicited by incubation with IL 22 or IL 11, originally derived from the pericryptal subset of gp38+ stromal cells and IFN *γ* supplied by natural killer cells [78].

### 2.7. *Clostridium difficile*

Similarly to *Salmonella enterica* sp., *Clostridium difficile* (*C. difficile*), a gram-positive, anaerobic, sporulating bacterium which accounts for a significant proportion of cases of antibiotics-associated diarrhea and pseudomembranous colitis, provokes cytoskeletal disarray by targeted inactivation of host cell Rho/Ras GTP-ases through its single-chain toxins *C. difficile* toxins (Cdt) A and B. Both toxins are equipped with a N-terminal glucosyltransferase and autoprotease domain which, after internalization and endosomal acidification, diffuse into the cytosol. Following autoproteolytic cleavage and release of glucosyltransferase, the Rho/Ras GTP-ase family members RhoA, Rac, and Cdc42 become mono-O-glycosylated and thereby inactivated, preventing them from interaction with their effectors possibly via steric hindrance [80]. Due to extensive involvement of the Rho/Ras GTP-ases in most actin-dependent processes, including stabilization of cell-cell contacts and cell shape-retaining stress fibers, any perturbation of this delicate switching element results in cell shrinkage, dissociation, and hence break-down of the intestinal barrier function. Furthermore, both toxins are able to induce apoptosis and pyrin inflammasome-induced pyroptosis [81–84]. A basic *in vitro* model of *C. difficile* infection was established using induced human intestinal organoids (iHIO) microinjected with toxigenic *C. difficile* strain VPI 10463 or nontoxigenic clinical isolate F200, respectively [85]. As expected, while infection with the latter did not result in a noticeable compromise of epithelial barrier function, inoculation with the toxin-producing strain caused apoptosis and severe disruption of the epithelium. Strikingly, separate microinjection of purified Cdt A into the iHIOs resulted in a profound redistribution of adherens and tight junction proteins as well as decomposition of actin filaments, exceeding the impact of Cdt B microinjection [85]. This observation contradicts former *in vitro* studies with intestinal cell lines, reporting an altogether higher potency for Cdt B [86–88]. However, in a mouse model of *C. difficile* colitis, rectal instillation of Cdt A alone triggered severe mucosal tissue damage and increased granulocyte infiltration as compared to Cdt B alone [89]. Arguably, these differences are related to the experimental conditions, with intestinal organoids being more likely to behave biologically like *in situ* tissue.

As far as incidence is concerned, the clinical severity and mortality rates of C. difficile infection seem to be inversely correlated to level of human serum albumin (HSA), which is considered a potential protective factor. Mechanistically, HSA is thought to bind Cdt A and Cdt B and therefore enhance auto-proteolytic cleavage, preventing toxin entry into the intestinal epithelial cell. Preliminary studies conducted by Di Masi et al. had resulted in a rapid decrease in serial transepithelial resistance measurements and cell viability of a Caco-2 monolayer culture exposed to a Cdt A-Cdt B mixture and CdtB alone, respectively [90]. By contrast, pretreatment with HSA was able to partially reverse the aforementioned effects and decrease the cellular uptake of Cdt B [90]. The same group was able to corroborate these findings using iPSC-derived human intestinal organoids generated by cellular reprogramming of keratinocytes from the plucked hair of a healthy human donor. After exposure to identical experimental conditions, intestinal organoids were assessed for macroscopic signs of structural disarray. These were reflected by the number of intact crypts as well as the distribution pattern of adherens junctions, which altogether pointed to a significantly diminished toxic effect associated with HSA [90]. Using the same *in vitro* model based on iPSC-derived intestinal organoids, Zhu et al. showed that the antibiotic bacitracin possesses TcdB-neutralizing properties translating into a reduction of TcdB-related glucosylation of Rac1 as well as reduced destruction of the filamentous actin cytoskeleton [91].

### 2.8. *Campylobacter jejuni*

Enteric microbial pathogens may not only cause infectious diseases of the intestinal tract but also have been linked with an increased risk of developing colorectal cancer. Malignant transformation can be achieved by promotion of an inflammatory environment, production of molecules affecting DNA stability, and alteration of proliferative responses [92]. Among others, *Campylobacter* sp., a common causative agent of food-borne infectious enteritis in industrial countries, is capable of synthesizing a genotoxin referred to as cytolethal distending toxin (CDT). This toxin is a ternary protein complex consisting of three subunits CDT A, B, and C, whereby CDT B acts as a DNase, inducing host DNA strand breaks. This critical role of CDT B was illustrated by the *in vitro* exposure of murine small intestinal organoids to bacterial lysates either from *Campylobacter jejuni* (*C. jejuni*) WT strain or *C. jejuni* containing a mutant CDT B allele [93]. In line with previous results derived from intestinal cell lines, incubation of intestinal organoids with lysates from the *C. jejuni* WT strain resulted in increased phosphorylation of histone H2AX, a marker for DNA damage, thus indicating elevated levels of DNA strand breaks [93].

## 3. Chances and Drawbacks of Intestinal Organoids

With the advent of the organoid technology, intestinal organoids have gained widespread acceptance as a validated and powerful platform to faithfully reflect the environmental conditions in the gut epithelium. A variety of source materials are suitable for efficiently generating intestinal organoids, ranging from adult multipotent to embryonic or reprogrammed pluripotent stem cells, all of which share the ability of self-renewal, directional expansion, and lineage commitment to differentiate into the principal cell types of the intestinal epithelium. Beyond that, pluripotent stem cells are competent to develop into any of the three germ layers (i.e., endoderm, mesoderm, and ectoderm) after exposure to spatially and temporally varying combinations and concentrations of growth factors. Therefore, intestinal organoids originating from pluripotent stem cells may additionally include mesodermal residues providing fibroblasts and smooth muscle cells, which have been shown to encase the organoids and support their morphogenesis via intimate epithelial-mesenchymal interactions [94, 95]. Undisputedly, organoids generated in such way will have a greater potential to resemble the complex cellular composition of the original tissue, allowing the role of the subepithelial stroma to be studied in the context of enteric invasive infections. In general, the use of intestinal organoids instead of a clonal cell line may prove advantageous in the context of scrutinizing transmission routes in which a specific cell type serves as the preferred site of invasion or provides a potential target structure for individual pathogens and their toxins, respectively. In this respect, intestinal organoids are also an inviting option for investigations of the gut epithelium-owned defense system mainly represented by the Paneth cell population. Paneth cells not only deliver antimicrobial substances for instantaneous neutralization of pathogens but also dynamically respond to infectious or inflammatory stimuli by undergoing hyperproliferation or de-differentiation into stem cells to replenish the Lgr5+ stem cell compartment and preserve epithelial integrity [96, 97]. We think that, owing to their unique genomic signature, intestinal organoids may theoretically be utilized for personalized studies to determine the individual susceptibility to certain toxins or toxin-producing pathogens. In several studies, individuals with non-blood group 0 have been predicted to be at higher risk of contracting diarrheal diseases caused by ETEC LT and CT, both of which rely on the basic sugar residue N-acetylgalactosamine for stable binding to the host cell membrane [98, 99]. In addition, pathogen- or toxin-treated intestinal organoids may be used to directly explore the efficacy of antitoxin agents by assessing and quantifying the residual cytotoxic impact on a naturally behaving population of primary intestinal cells. Planar arrays of human colon-derived organoids fused with automated imaging and analysis tools have already yielded promising results which may in future enable large-scale screening of toxic compounds and drugs, respectively [100].

However, intestinal organoids do not come without shortcomings. The host's defensive capacities are not confined to the intestinal epithelium itself but are equally dependent on the resident microbial community of the gut. In a homeostatic ecosystem, the highly diversified commensal microbiome hedges enteropathogenic colonization of the mucosal surface through a mechanism termed “colonization resistance.” Mainly due to a limited nutrient supply, resident microbiota constantly compete with invading pathogens to prevail against the occupation of available nutrient niches and thus prevent their uncontrolled spreading. Not only by inhabitation of the gut lumen itself but also by excretion of metabolic waste products does the commensal microbiome efficiently contribute to containing enteropathogens and at the same time fortifying the intestinal epithelial barrier. Under the influence of *Bifidobacteria*, *Lactobacilli*, and *Firmicutes* sp., complex carbohydrates are broken down into short chain fatty acids (SFA) which have been reported to promote colonization resistance [101, 102]. Exposure of intestinal organoids to the SFA butyrate, propionate, and acetate was significantly associated with a promotion of epithelial proliferation and cell turnover for each single agent, with an additive effect being observed for a mixture of all three SFA [103]. So far, the microbiome as a critical protective factor has only been inadequately reflected by organoid-based enteric infection models for a number of reasons. Given that the intestinal luminal content comprises trillions of commensal microbes accounting for an estimated 500-1,000 bacteria species [104], a selection of a manageable number (usually 1-2 according to the literature) of microbial species to be incorporated into the organoids is rather insufficient. Another hurdle is imposed by the standard aerobic culturing conditions of organoids precluding the propagation of obligate anaerobic commensal microbiota. Therefore, only oxygen-tolerant commensal bacterial species such as *Akkermansia muciniphila*, *Faecalibacterium prausnitzii* [105], *Escherichia coli* [55], and *Bacteroides thetaiotaomicron* [106] have been successfully used to colonize intestinal organoids. This essential limitation has been recognized and addressed by modification of the microfluidic gut-on-a-chip technology to create an anoxic-oxic interface resembling the colonic mucosa. This permits a stable cultivation of the obligate anaerobic commensal bacteria *Bifidobacterium adolescentis* and *Eubacterium hallii*, respectively, in direct contact with an intestinal epithelial monolayer [107]. As opposed to establishing optimal growth conditions for anaerobes, bacterial growth within inoculated intestinal organoids is to be restricted to the luminal space by the utilization of antibiotic-containing media and the selection of microbial strains according to their individual resistance. Besides the microbiota, the intestinal luminal content carries an abundance of nutrients provided by dietary ingredients and endogenous metabolites, mucus, and bile acids, all of which have been shown to affect host defense response to a variable extent but are only poorly recapitulated by intestinal organoids.

Intestinal organoids have impressively demonstrated their ability to serve as a resource for the advanced *in vitro* modeling of enteric infections. Although this review is primarily dedicated to outlining current bacteriological knowledge acquired from infected intestinal organoids, it is noteworthy that analogous disease models exist for various parasitic [108, 109] and viral pathogens [110, 111] of the gastrointestinal tract. In fact, in light of the ongoing COVID-19 pandemic, attention has been shifted to employing organoid technology to help reveal fundamental mechanisms of viral entry and intracellular replication. In particular, human intestinal organoids play a pivotal role in supporting a robust replication of formerly unculturable viral agents such as the human norovirus [111], extending their utility for future SARS-CoV-2-related pathogenetic studies and high-throughput therapeutic drug screening. Considering the presumable zoonotic background of SARS-CoV-2, researchers have for the first time established intestinal organoids from Chinese horseshoe bats suspected to be one of the natural reservoirs [112]. With the intestinal organoid culturing protocol also being applicable to other non-human mammalian species such as the cow [113], pig [113, 114], dog [115], and cat [116], important strides have been made to consolidate our current pathophysiological understanding of zoonotic diseases. Combining the findings derived from intestinal organoids spanning different species will certainly be of added value for characterizing a broad spectrum of common zoonotic bacterial pathogens affecting the intestinal tract. Prospectively, among the numerous advantages related to intestinal organoids, preterm recognition of potentially human relevant microorganisms and expeditious *in vitro* screening of promising drug candidates might become a key application in opposing zoonotic bacterial diseases with life-threatening potential.

## Figures and Tables

**Table 1:** 

Causative agent	Source and characteristics of infected organoid culture system	Findings/ objectives	References
Campylobacter jejuni	(i) Adult murine small intestinal organoids	(i) Infection-related genotoxicity (induction of DNA strand breaks)	(i) [86]
Clostridium difficile	(i) iPSC-derived human intestinal organoids (ii) iPSC-derived human intestinal organoids	(i) Attenuation of Cdt B-induced cytopathic effect by exposure to HSA (ii) Neutralization of TcdB-induced cytoskeletal disarray by bacitracin	(i) [83] (ii) [84]
Enterohemorrhagic Escherichia coli	(i)Adult human colon-derived organoids (ii) ESC-derived human intestinal organoids supplemented with PMN	(i) EspP-mediated proteolytic degradation of protocadherin 24, effacement of microvillar bridges of the enterocytic brush border; EspP equipped with enterotoxin-like properties (ii) *In vitro* modeling of EHEC enteric invasive infection	(i) [44] (ii) [49]
Enterotoxinogenic Escherichia coli	(i) Adult human small intestinal organoids (ii) Adult human small intestinal organoids supplemented with monocyte-derived macrophages	(i) Preferential adhesion to blood group A-glycosylated epithelial surfaces via lectin EtpA (ii) Mitigation of bacteria-stimulated inflammation and intestinal barrier dysfunction by resident macrophages	(i) [30] (ii) [31]
Listeria monocytogenes	(i) Fetal tissue-derived human intestinal organoids (ii)Adult human small intestinal “basal-out” organoids (iii) Adult murine intestinal organoids	(i) Goblet cells as preferred cell type of entry (ii) Enforced endocytotic uptake via basolateral cellular target structures E-cadherin and Met (iii) Accelerated epithelial renewal and reduction in goblet cell numbers mediated by STAT1 and STAT3	(i) [67] (ii) [59] (iii) [72]
Salmonella spcc.	(i) Adult human small intestinal organoids (ii) Adult murine small intestinal “apical-out” organoids (iii) Adult human ileum-derived organoids (iv) Adult murine small intestinal organoids (v) Adult murine small intestinal organoids supplemented with probiotic L. acidophilus	(i) Inducible transdifferentiation of enterocytes into M cells as favored portal of entry (ii) Preference of apical transmission route; luminal shedding of infected enterocytes (iii) Invasion and intracellular dispersion dependent on exploitation of host cell cytoskeleton (iv) Disintegration of epithelial Zonula occludens, enhanced NF-*κ*B signaling; upregulation of goblet cell gene markers (v) Reversal of infection-induced upregulation of Wnt3 and Toll-like receptor 2 and 4	(i) [54] (ii) [59] (iii) [60] (iv) [61, 62] (v) [63]
Shigella flexneri	(i) Adult human colon-derived organoids (ii) Adult human ileum-derived organoids pretreated with TNF *α* to amplify M cell population (iii) Adult human colon-derived organoids (iv) Adult human intestinal organoids	(i) Production of adhesive biofilm dependent on luminal exposure to glucose and bile salts (ii) Preferred host cell invasion via M cell transcytosis, enhanced NF-*κ*B signaling; upregulation of MUC2 expression (iii) Polymerization of actin fibers to facilitate intracellular trafficking; enhanced NF-*κ*B signaling (iv) Anti-infective effectiveness of bacteriophages	(i) [37] (ii) [38] (iii) [39] (iv) [41]
Vibrio cholerae	(i) Swelling assay conducted in human adult rectum-derived organoids (ii) iPSC-derived human intestinal organoids	(i) Neutralization of CT by monovalent and multivalent metanitrophenyl *α*-galactoside-bound polymers (ii) *In vitro* modeling of cholera enteric infection	(i) [21] (ii) [23]
